# Upregulation of miR-150-5p alleviates LPS-induced inflammatory response and apoptosis of RAW264.7 macrophages by targeting Notch1

**DOI:** 10.1515/biol-2020-0058

**Published:** 2020-07-28

**Authors:** Xiaoyan Deng, Zhixing Lin, Chao Zuo, Yanjie Fu

**Affiliations:** Department of ICU, The First Affiliated Hospital of Hainan Medical University, Haikou, Hainan Province, China; Shandong Medical College, Jinan, Shandong Province, China; Department of Burn and Plastic Surgery, Linyi People’s Hospital, No. 27, Jiefang Rd, Linyi City, 276000, Shandong Province, China

**Keywords:** sepsis, miR-150-5p, Notch1, inflammatory response, apoptosis

## Abstract

Circulating miR-150-5p has been identified as a prognostic marker in patients with critical illness and sepsis. Herein, we aimed to further explore the role and underlying mechanism of miR-150-5p in sepsis. Quantitative real-time-PCR assay was performed to detect the expression of miR-150-5p upon stimulation with lipopolysaccharide (LPS) in RAW264.7 cells. The levels of tumor necrosis factor-α, interleukin (IL)-6 and IL-1β were measured by ELISA assay. Cell apoptosis was determined using flow cytometry. Western blot was used to assess notch receptor 1 (Notch1) expression in LPS-induced RAW264.7 cells. Dual-luciferase reporter assay was employed to validate the target of miR-150-5p. Our data showed that miR-150-5p was downregulated and Notch1 was upregulated in LPS-stimulated RAW264.7 cells. miR-150-5p overexpression or Notch1 silencing alleviated LPS-induced inflammatory response and apoptosis in RAW264.7 cells. Moreover, Notch1 was a direct target of miR-150-5p. Notch1 abated miR-150-5p-mediated anti-inflammation and anti-apoptosis in LPS-induced RAW264.7 cells. miR-150-5p alleviated LPS-induced inflammatory response and apoptosis at least partly by targeting Notch1 in RAW264.7 cells, highlighting miR-150-5p as a target in the development of anti-inflammation and anti-apoptosis drugs for sepsis treatment.

## 1 Introduction

Sepsis, a huge and expensive medical problem around the world, affects more than 19 million people every year and has become the leading cause of death in critically ill patients [1]. Sepsis is characterized by the dysregulation of inflammation following primary bacterial infection and accompanied with systemic inflammatory response syndrome (SIRS) [2]. Lipopolysaccharide (LPS) has been postulated to trigger SIRS and is well known as an important mediator of sepsis [3]. During the inflammatory response, activated macrophages and neutrophils generate prodigious amounts of inflammatory factors, such as tumor necrosis factor (TNF)-α, interleukin (IL)-6 and IL-1β [4]. An excessive inflammatory response is autodestructive and may lead to microcirculatory dysfunction, causing meningitis, constrictive pericarditis, arthralgia, other organ damage, septic shock and death [5]. Therefore, it is imperative to identify more effective molecular targets for protecting against dysregulated inflammation and helping to control inflammation.

MicroRNAs (miRNAs), a type of evolutionarily conserved small noncoding RNAs with ∼22 nucleotides in length, function as key molecular components of the cell in both normal and pathologic states [6]. Mature miRNAs negatively regulate gene expression by binding to the 3′-untranslated region (UTR) of target mRNAs, leading to translational repression and target mRNA degradation [7]. miRNAs have been identified as regulators of the immune response, with potentially translational implications in sepsis [8,9]. Downregulation of miR-150 (also called miR-150-5p) was found in plasma samples of sepsis patients, and circulating miR-150 was identified as a prognostic marker in patients with critical illness and sepsis [10,11,12]. Moreover, miR-150 was reported to be involved in the pathogenesis of sepsis [13,14]. A recent document demonstrated that miR-150 repressed LPS-induced inflammatory factors and apoptosis by regulating NF-κB1 in human umbilical vein endothelial cells [15]. Additionally, LPS infusion into healthy humans led to decreased miR-150 expression in peripheral blood leukocytes [16]. Herein, we aimed to further explore the role and underlying mechanism of miR-150-5p in sepsis.

In this study, our data supported that miR-150-5p expression was reduced by LPS and miR-150-5p alleviated LPS-induced inflammatory response and apoptosis in RAW264.7 cells. Notch receptor 1 (Notch1) was identified as a direct target of miR-150-5p. Moreover, miR-150-5p alleviated LPS-induced inflammatory response and apoptosis at least partly by targeting Notch1 in RAW264.7 cells.

## 2 Materials and methods

### 2.1 Cell culture and treatment

Murine macrophage cell line RAW264.7 was purchased from ATCC (Manassas, VA, USA) and cultured in Dulbecco’s Modified Eagle’s medium (Gibco, Big Cabin, OK, USA) containing 10% fetal bovine serum (HyClone, GE Healthcare, Logan, UT, USA) and 1% penicillin/streptomycin (Life Technologies, Carlsbad, CA, USA) at 37°C in a 5% CO_2_ incubator.

Upon reaching a confluence of 70–80%, RAW264.7 cells were exposed to LPS (*Escherichia coli* serotype; Sigma-Aldrich, St. Louis, MO, USA) with a final concentration of 1 µg/mL for 24 h [17].

### 2.2 Cell transfection

The commercial modified miR-150-5p agonist (agomiR-150-5p, 5′-GUGACCAUGUUCCCAACCCUCU-3′) and negative control of agomiR (agomiR-NC, 5′-UUCUCCGAACGUGUCACGUTT-3′), miR-150-5p antagonist (antagomiR-150-5p, 5′-AGAGGGUUGGGAACAUGGUCAC-3′) and negative control of antagomiR (antagomiR-NC, 5′-CAGUACUUUUGUGUAGUACAA-3′), siRNA targeting Notch1 (si-Notch1, 5′-UCGCAUUGACCAUUCAAACUGGUGG-3′) and negative control of siRNA (si-NC, 5′-UUCUCCGAACGUGUCACGUTT-3′), Notch1 (accession number: JQ303092.1) overexpression vectors (pcDNA-Notch1, Notch1 sequence was cloned into pcDNA3.1 plasmid with BamH I and Xho I sites) and negative control pcDNA-NC were designed and synthesized by GenePhama (Shanghai, China). Upon reaching 60–70% confluence, RAW264.7 cells were transiently transfected with the indicated oligonucleotide (10 nM) and/or plasmid (10 ng) using Lipofectamine 2000 transfection reagent (Thermo Fisher Scientific, San Jose, CA, USA) according to the manufacturer’s recommendations.

### 2.3 RNA isolation and quantitative real-time PCR (qRT-PCR) of miR-150-5p

Total RNA was isolated from cells with the miRNeasy Mini Kit (Qiagen, Valencia, CA, USA) according to the manufacturer’s protocol. RNA (50 µg) was reverse transcribed into cDNA using TaqMan Reverse Transfection kit (Applied Biosystems, Foster city, CA, USA), and qRT-PCR was performed using TaqMan MicroRNA Assay kit (Applied Biosystems) on the 7500 Fast Real-Time PCR system (Applied Biosystems). The relative expression of miR-150-5p was normalized using U6 expression and the 2^−ΔΔCt^ method. The following primers were used: 5′-TCTCCCAACCCTTGTACCAGTG-3′ (miR-150-5p, sense), 5′-GTGCGTGTCGTGGAGTC-3′ (miR-150-5p, antisense), 5′-GCTTCGGCAGCACATATACTAA-3′ (U6, sense) and 5′-AACGCTTCACGAATTTGCGT-3′ (U6, antisense).

### 2.4 ELISA assay for TNF-α, IL-6 and IL-1β measurement

The levels of TNF-α, IL-6 and IL-1β in treated cells were measured using ELISA kits (R&D Systems Europe Ltd, Abingdon, UK) according to the manufacturer's instruction. Briefly, cell samples (10 µL/well) were added to 96-well plates containing capture antibodies specific to TNF-α, IL-6 and IL-1β, and then HRP-conjugated reagent (100 µL/well) was added to each well. After 1 h incubation at 37°C, the plates were washed with washing buffer (1×), followed by the incubation with a substrate solution. The absorbance at 450 nm was determined by a Spectra-Max300 microplate reader (Molecular Devices, Sunnyvale, CA, USA).

### 2.5 Flow cytometry for apoptosis measurement

Cell apoptosis was evaluated by flow cytometry using Annexin V/FITC Apoptosis Detection Kit (BD Biosciences, Franklin Lakes, NJ, USA) according to the manufacturer's recommendations. Briefly, cells were resuspended with 1× binding buffer and then were stained with Annexin V-FITC and PI. After 15 min incubation at room temperature, the data were analyzed by a FACSCalibur flow cytometer (BD Biosciences) with Cell Quest Pro software. Cells that were stained with Annexin V-FITC (Q2) and Annexin V-FITC together with PI (Q4) were designated as early and late apoptotic cells, respectively.

### 2.6 Dual-luciferase reporter assay

Online software miRcode (http://www.mircode.org/) and miRBase (http://www.mirbase.org/) were used to predict the targets of miR-150-5p. The segment of Notch1 3′-UTR containing the complementary sites of miR-150-5p and mismatched miR-150-5p-binding sites were cloned into pmirGLO plasmid (Promega, Mannheim, Germany), to generate Notch1 3′-UTR wild-type reporter vector (WT-Notch1) and mutant-type reporter vector (MUT-Notch1), respectively. RAW264.7 cells were cotransfected with 100 ng of WT-Notch1 or MUT-Notch1 and 10 nM of agomiR-150-5p. The relative luciferase activity was determined by using the dual-luciferase reporter assay system (Promega).

### 2.7 Western blot for Notch1 detection

Total protein was extracted from cells using RIPA lysis buffer (50 mM Tris–HCl pH = 7.4, 150 mM NaCl, 1% Triton X-100, 0.1% SDS, 0.5% sodium deoxycholate and 1 mM phenylmethylsulfonyl fluoride) supplemented with protease inhibitor cocktail (Sigma-Aldrich) and quantified with a BCA protein assay kit (Thermo Fisher Scientific). A total of 50 µg of protein samples were separated by 10% SDS-PAGE gels and then transferred onto the polyvinylidene difluoride membranes (Life Technologies). After being blocked with 5% non-fat milk, the membranes were probed with primary antibodies against Notch1 (1:1,000; Cell Signaling Technology, Danvers, MA, USA) and GAPDH (1:1,000; Cell Signaling Technology), followed by the incubation with horseradish peroxidase-conjugated secondary antibody (1:1,000; Cell Signaling Technology). The protein bands were visualized using Pierce ECL Western Blotting Substrate (Thermo Fisher Scientific) and quantified by the ChemiStage CC-16 mini imaging system (Kurabo, Osaka, Japan).

### 2.8 Statistical analyses

Statistical analyses were performed using GraphPad Prism 5.0 software (GraphPad Software Inc., CA, USA). All data were reported as mean ± SEM from at least three independent experiments. Differences between two groups were compared using a Student’s *t*-test, and analysis of variance was used for the comparison between multiple groups. A *P* value less than 0.05 was considered statistically significant.

## 3 Results

### 3.1 miR-150-5p expression was reduced by LPS in RAW264.7 cells

**Figure 1:**
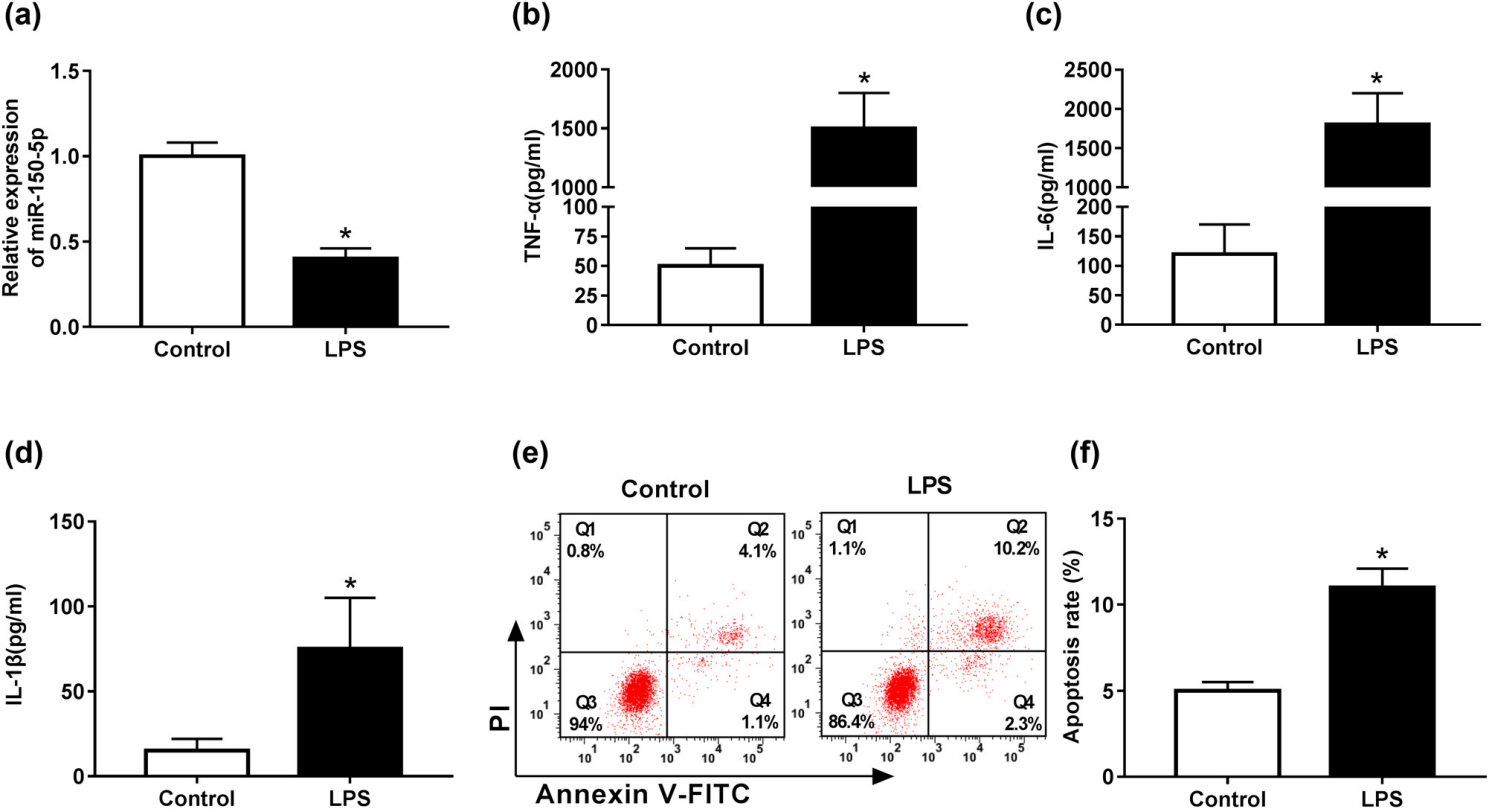
miR-150-5p expression was reduced by LPS in RAW264.7 cells. RAW264.7 cells were stimulated with 1 µg/mL LPS for 24 h, followed by the detection of (a) miR-150-5p expression by qRT-PCR, (b–d) TNF-α, IL-6 and IL-1β levels by ELISA assays, and (e and f) cell apoptosis rate by flow cytometric analysis with Annexin V-FITC/PI staining. *P < 0.05 vs control.

First, we validated how LPS influenced miR-150-5p expression in RAW264.7 cells. As shown in Figure 1a, LPS stimulation in RAW264.7 cells resulted in a significant decrease in miR-150-5p expression compared with negative control. Then, we determined the role of LPS in cell inflammatory response and apoptosis in RAW264.7 cells. Results revealed that in comparison to their counterparts, LPS stimulation led to a strong increase in TNF-α, IL-6 and IL-1β levels, indicating LPS-induced inflammatory response of RAW264.7 cells (Figure 1b–d). Moreover, cell apoptosis was highly elevated (from 5.2% to 12.5%) by LPS in RAW264.7 cells compared with normal control (Figure 1e and f).

### 3.2 miR-150-5p alleviated LPS-induced inflammatory response and apoptosis in RAW264.7 cells

**Figure 2:**
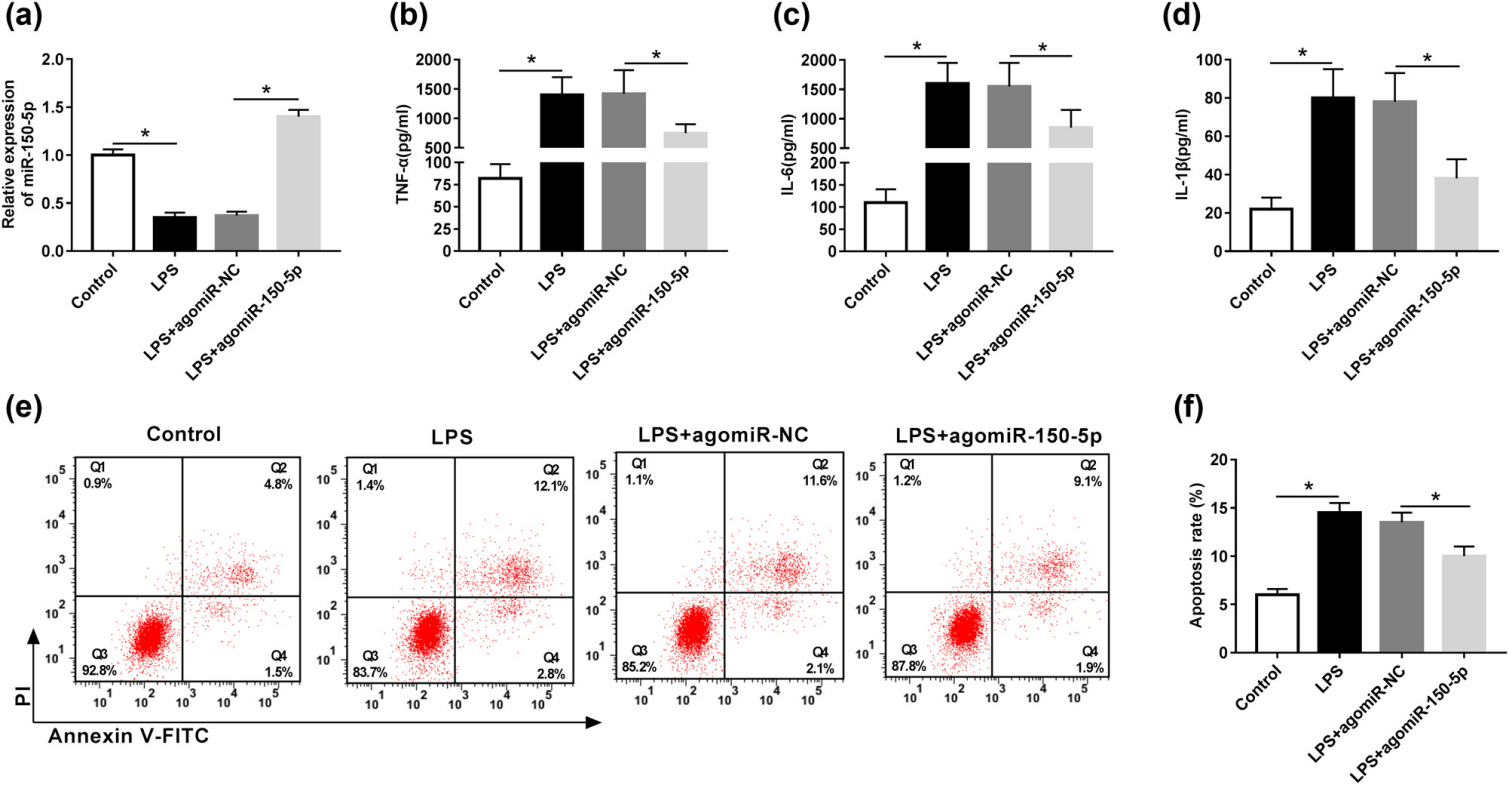
miR-150-5p alleviated LPS-induced inflammatory response and apoptosis in RAW264.7 cells. RAW264.7 cells were transfected with agomiR-150-5p or agomiR-NC and then were stimulated with 1 µg/mL LPS for 24 h, followed by the determination of (a) miR-150-5p expression by qRT-PCR, (b–d) TNF-α, IL-6 and IL-1β levels by ELISA assays, and (e and f) cell apoptosis by flow cytometric analysis with Annexin V-FITC/PI staining. *P < 0.05 vs control or LPS + agomiR-NC.

To explore the role of miR-150-5p in LPS-induced inflammatory response and apoptosis in RAW264.7 cells, we manipulated miR-150-5p expression by transfecting agomiR-150-5p into RAW264.7 cells prior to LPS stimulation. As shown in Figure 2a, agomiR-150-5p transfection resulted in about 3.8-fold increase in miR-150-5p expression in LPS-induced RAW264.7 cells compared with agomiR-NC control. Then, ELISA results indicated that miR-150-5p overexpression significantly alleviated LPS-induced inflammatory response, as presented by a decrease in TNF-α, IL-6 and IL-1β levels in RAW264.7 cells (Figure 2b–d). Moreover, LPS-induced apoptosis was evidently attenuated (from 13.7% to 11.0%) by miR-150-5p upregulation when compared with negative control (Figure 2e and f).

### 3.3 Notch1 was a direct target of miR-150-5p

**Figure 3:**
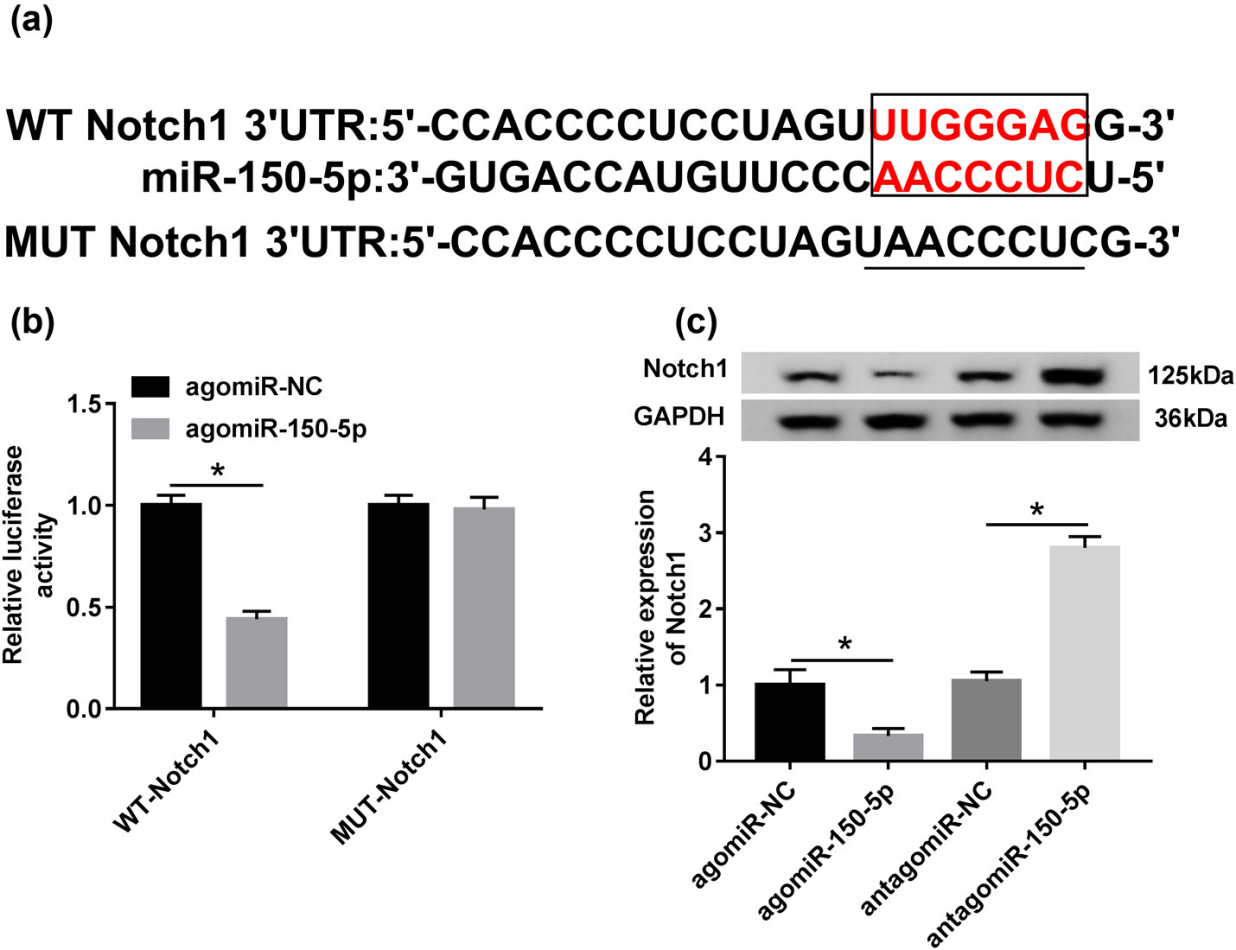
Notch 1 was a direct target of miR-150-5p. (a) The sequences in the 3′-UTR region of Notch1 targeted by miR-150-5p and its mutant sequences in the seed region. (b) The relative luciferase activity of WT-Notch1 or MUT-Notch1 was measured in agomiR-NC- or agomiR-150-5p-transfected RAW264.7 cells. (c) Notch1 expression was determined in agomiR-NC, agomiR-150-5p, antagomiR-NC or antagomiR-150-5p-transfected RAW264.7 cells by western blot analysis. *P < 0.05 vs agomiR-NC.

**Figure A1:**
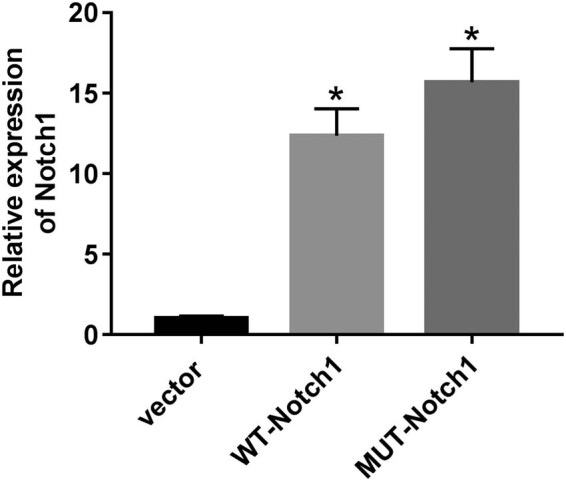
The transfection efficiency of WT-Notch1 and MUT-Notch1 was assessed by qRT-PCR.

To further understand the role of miR-150-5p, we used miRcode and miRBase online software to help identify its molecular targets. The predicted data revealed that Notch1 contained a target region that matches to miR-150-5p (Figure 3a). To verify this, dual-luciferase reporter assays were performed. Notch1 3′-UTR wild-type reporter vectors containing the complementary sites of miR-150-5p (WT-Notch1) and the mutant-type vectors in the seed region (MUT-Notch1) were transfected into RAW264.7 cells, along with agomiR-150-5p or agomiR-NC. The data of qRT-PCR showed that the level of Notch1 was prominently elevated in WT-Notch1- and MUT-Notch1-transfected RAW264.7 cells, demonstrating the high transfection efficiency (Figure A1). Moreover, the luciferase activity of WT-Notch1 was markedly repressed by agomiR-150-5p (Figure 3b). However, little change was observed in luciferase of MUT-Notch1 in the presence of agomiR-150-5p (Figure 3b). We then observed whether Notch1 expression was influenced by miR-150-5p. Western blot data demonstrated that Notch1 expression was significantly decreased by agomiR-150-5p, while it was remarkably increased when miR-150-5p depleted (Figure 3c).

### 3.4 Notch1 silencing ameliorated LPS-induced inflammatory response and apoptosis in RAW264.7 cells

**Figure 4:**
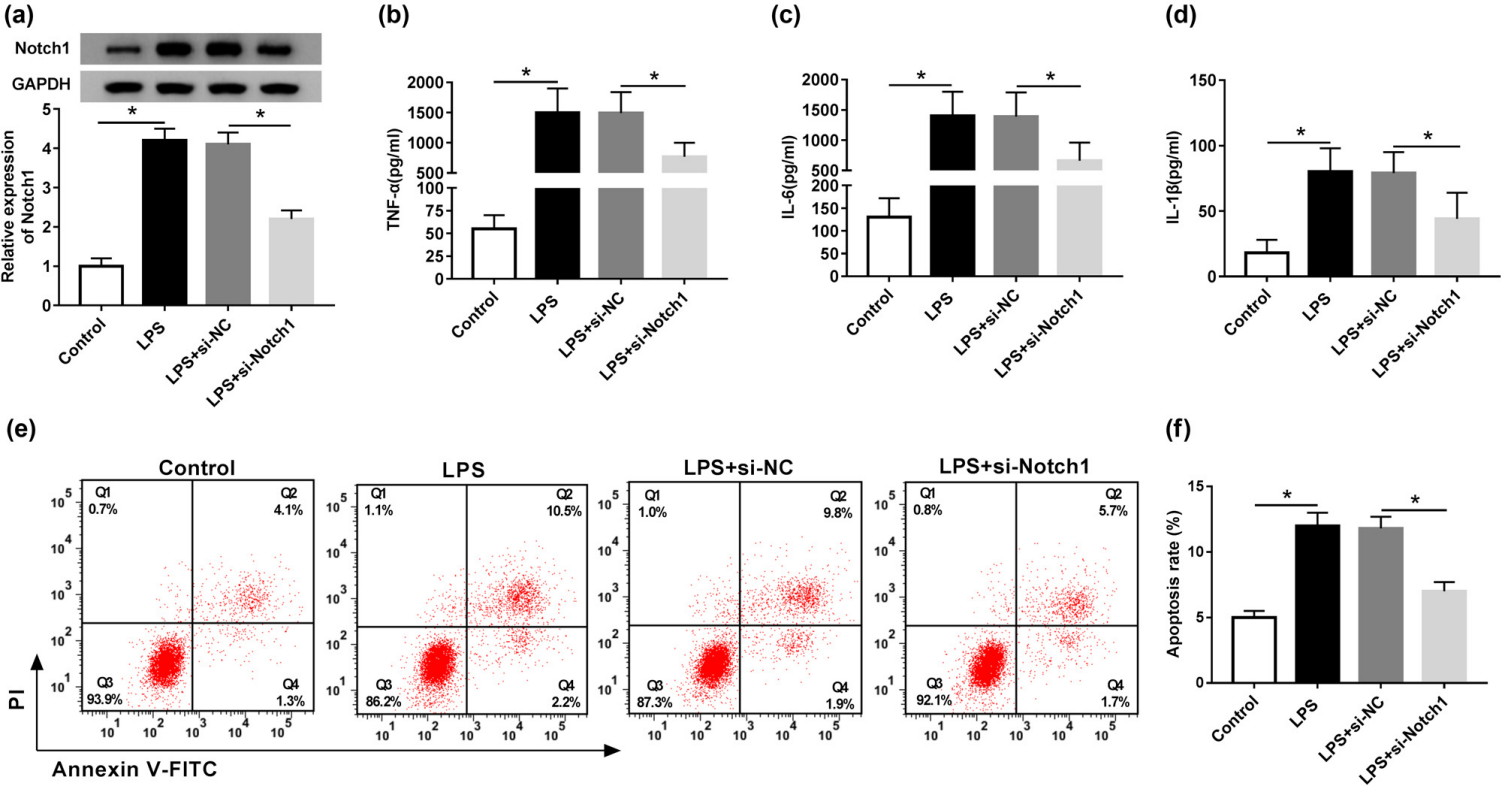
Notch1 silencing ameliorated LPS-induced inflammatory response and apoptosis in RAW264.7 cells. RAW264.7 cells were transfected with si-Notch1 or si-NC prior to LPS stimulation, followed by the measurement of (a) Notch1 level by western blot analysis, (b–d) TNF-α, IL-6 and IL-1β levels by ELISA assays, and (e and f) cell apoptosis by flow cytometric analysis. *P < 0.05 vs control or LPS + si-NC.

Then, we identified whether LPS affected the Notch1 level in RAW264.7 cells. As shown in Figure 4a, the Notch1 level was substantially increased after 24 h LPS stimulation in RAW264.7 cells. To further investigate the role of Notch1 in LPS-induced inflammatory response and apoptosis, RAW264.7 cells were transfected with si-Notch1 or si-NC prior to LPS treatment. Results demonstrated that compared with negative control, si-Notch1 transfection led to a decrease in Notch1 expression in LPS-induced RAW264.7 cells (Figure 4a). ELISA data indicated that Notch1 silencing ameliorated LPS-induced inflammatory response in RAW264.7 cells in comparison to the control group (Figure 4b–d). Moreover, LPS-induced apoptosis of RAW264.7 cells was highly reversed (from 11.7% to 7.4%) by Notch1 silencing (Figure 4e and f).

### 3.5 Notch1 overexpression abated miR-150-5p-mediated anti-inflammation and anti-apoptosis in LPS-induced RAW264.7 cells

**Figure 5:**
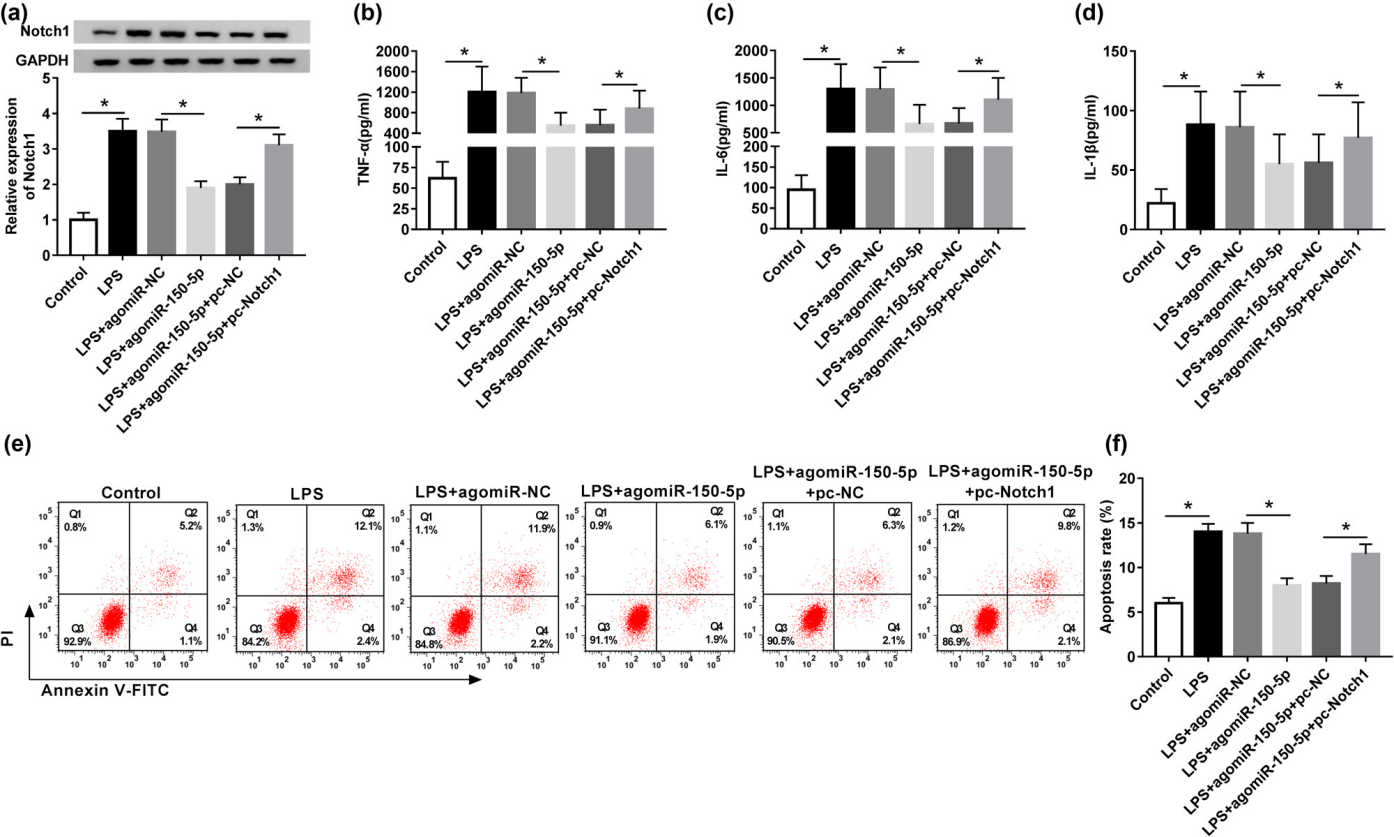
miR-150-5p exerted its regulatory effect on inflammatory response and apoptosis in LPS-induced RAW264.7 cells by Notch1. RAW264.7 cells were transfected with agomiR-NC, agomiR-150-5p, agomiR-150-5p + pcDNA-NC or agomiR-150-5p + pcDNA-Notch1 prior to LPS stimulation. (a) Western blot analysis of Notch1 expression in treated cells. (b–d) ELISA assays of TNF-α, IL-6 and IL-1β levels in treated cells. (e and f) Flow cytometric analysis of cell apoptosis in treated cells. *P < 0.05 vs control or LPS + agomiR-NC or LPS + agomiR-150-5p + pc-NC.

To provide further mechanistic insight into the link between miR-150-5p and Notch1 on inflammatory response and apoptosis in LPS-induced RAW264.7 cells, RAW264.7 cells were cotransfected with agomiR-150-5p and pcDNA-Notch1 prior to LPS treatment. Western blot analysis revealed that agomiR-150-5p-mediated inhibition of Notch1 expression was dramatically abated by pcDNA-Notch1 cotransfection in LPS-induced RAW264.7 cells (Figure 5a). Subsequent results showed that the suppression effect of miR-150-5p on inflammatory response and apoptosis was significantly reversed by Notch1 overexpression in LPS-induced RAW264.7 cells (Figure 5b–f).

## 4 Discussion

miRNAs play a crucial role in inflammatory response and have been demonstrated to be involved in sepsis [18]. Recently, a series of circulating miRNAs have been identified as potential diagnosis biomarkers in sepsis, such as miR-146a and miR-223 [19], miR-15a and miR-16 [20], and miR-25 [21]. Moreover, miR-30a was demonstrated to suppress IL-10-induced cytokine release via regulating STAT1-MD-2 in monocytes of sepsis [22]. miR-23b repressed the secretion of inflammatory factors in sepsis [8]. Conversely, miR-15a/16 reduced the survival of septic mice by repressing phagocytosis and bacterial clearance [23]. Besides, miR-155 expression and inflammatory factors were elevated in the mice with LPS-induced sepsis and high expression of miR-155 implicated in the pathological processes of sepsis [24].

LPS is widely accepted to trigger the secretion of pro-inflammatory cytokines in human leukocytes, leading to the dysregulation of the transcriptome, including miRNA expression [25]. miR-150 expression was reported to be decreased in peripheral blood leukocytes during acute LPS-induced inflammation and in plasma samples of sepsis patients [10,16]. In the present study, we validated that miR-150-5p was downregulated in LPS-stimulated RAW264.7 cells, similar to previous studies [11,16]. Furthermore, our data indicated that miR-150-5p alleviated LPS-induced inflammatory response and apoptosis of RAW264.7 cells. Consistent with our findings, Ma et al. [15] manifested that miR-150 inhibited inflammatory response and apoptosis in LPS-treated human umbilical vein endothelial cells by targeting NF-κB1.

miRNAs have been postulated to exert their functions mainly by negatively regulating the expression of endogenous target genes [7]. Herein, we used online software miRcode and miRBase to predict the targets of miR-150-5p. Among these predicted candidates, Notch1 was of interest in the present study, considering its important role in inflammatory response [26,27]. Subsequently, we confirmed that Notch1 was a direct target of miR-150-5p in RAW264.7 cells. Notch1 deficiency was reported to inhibit inflammatory response by regulating the expression levels of vascular endothelial growth factor receptor-1 and inflammatory cytokines in macrophages [26,28]. Notch1 contributed to an amplification of inflammatory response in LPS-induced macrophages by upregulating the NF-κB activity [29]. More interestingly, Notch1 was reported to be implicated in the pathogenesis of sepsis [30,31]. In this study, our data indicated an increase in Notch1 expression in LPS-stimulated RAW264.7 cells, in accordance with previous studies [29,32]. Subsequently, our data supported that Notch1 silencing ameliorated LPS-induced inflammatory response and apoptosis in RAW264.7 cells.

In this report, for the first time, we identified that Notch1 abated miR-150-5p-mediated anti-inflammation and anti-apoptosis in LPS-induced RAW264.7 cells. Similarly, Jiang et al. [33] elucidated that high miR-34a expression resulted in decreased inflammatory response in LPS-induced murine macrophages by targeting Notch1. Wang et al. [34] found that miR-146b protected cardiomyocytes against sepsis-induced inflammatory response and apoptosis by the repression of Notch1.

In conclusion, we first identified that miR-150-5p exerted anti-inflammation and anti-apoptosis function at least partly by targeting Notch1 in LPS-induced RAW264.7 macrophages, highlighting miR-150-5p as a target in the development of anti-inflammation and anti-apoptosis drugs for sepsis treatment.
